# In vitro effects of nanoparticles on renal cells

**DOI:** 10.1186/1743-8977-5-22

**Published:** 2008-12-19

**Authors:** Béatrice L'Azou, Joana Jorly, Dinhill On, Elisabeth Sellier, Frédéric Moisan, Jocelyne Fleury-Feith, Jean Cambar, Patrick Brochard, Céline Ohayon-Courtès

**Affiliations:** 1 Laboratoire Santé – Travail – Environnement, EA 3672, Université de Bordeaux, Victor Segalen Bordeaux 2,146 rue Léo-Saignat, 33 076 Bordeaux Cedex, France; 2 CREMEM Centre de Ressources en Microscopie Electronique et Microanalyse, Université de Bordeaux, Avenue des Facultés, 33 405 Talence, France; 3 Laboratoire d'Etude des Particules Inhalées, 11-13 rue Georges Eastman, 75103 Paris, France

## Abstract

**Background:**

The ability of nanoparticles to cross the lung-blood barrier suggests that they may translocate to blood and to targets distant from their portal of entry. Nevertheless, nanotoxicity in organs has received little attention. The purpose of this study was to evaluate nanotoxicity in renal cells using *in vitro *models. Various carbon black (CB) (FW2–13 nm, Printex60-21 nm and LB101-95 nm) and titanium dioxide (TiO_2_-15 and TiO_2_-50 nm) nanoparticles were characterized on size by electron microscopy. We evaluated theirs effects on glomerular mesangial (IP15) and epithelial proximal tubular (LLC-PK_1_) renal cells, using light microscopy, WST-1 assay, immunofluorescence labeling and DCFH-DA for reactive oxygen species (ROS) assay.

**Results:**

Nanoparticles induced a variety of cell responses. On both IP15 and LLC-PK_1 _cells, the smallest FW2 NP was found to be the most cytotoxic with classic dose-behavior. For the other NPs tested, different cytotoxic profiles were found, with LLC-PK_1 _cells being more sensitive than IP15 cells. Exposure to FW2 NPs, evidenced in our experiments as the most cytotoxic particle type, significantly enhanced production of ROS in both IP15 and LLC-PK_1 _cells. Immunofluorescence microscopy using latex beads indicated that depending on their size, the cells internalized particles, which accumulated in the cell cytoplasm. Additionally using transmission electronic microscope micrographs show nanoparticles inside the cells and trapped in vesicles.

**Conclusion:**

The present data constitute the first step towards determining *in vitro *dose effect of manufactured CB and TiO_2 _NPs in renal cells. Cytotoxicological assays using epithelial tubular and glomerular mesangial cell lines rapidly provide information and demonstrated that NP materials exhibit varying degrees of cytotoxicity. It seems clear that *in vitro *cellular systems will need to be further developed, standardized and validated (relative to *in vivo *effects) in order to provide useful screening data about the relative toxicity of nanoparticles.

## Background

Nanotechnology can be defined as the techniques aimed at characterizing and producing materials on the nanometer scale (< 100 nm) and exhibiting specific physical/chemical properties and functions. Nanoparticles (NPs) are currently commercially manufactured and can be carbon- or metal-based materials (quantum-dots, nanogold, metal oxides), dendrimers and composites. Carbon nanoparticles will be produced in tons and consequently will lead to increased human and environmental exposure due to normal use, fugitive emissions, accidental spills and disposal of materials after use. Despite their wide application, little is known about their human health and environmental implications.

Numerous epidemiological studies have associated exposure to small particles such as combustion-generated fine particles with lung cancer, heart disease, asthma and/or increased mortality. Both Donalson et al., [1,2] and Oberdörster [3] concluded in reviews that ultrafine particles of low-solubility and low toxicity materials are more inflammogenic in the rat lung than larger particles of the same material. Additionally, NPs are able to penetrate deeply into the respiratory tract. Once deposited in the alveolar region, they may translocate to blood and to sites distant from their portal of entry such as the liver, spleen, kidney and brain [3-8]. Their migration to distant sites is an important issue with regard to their toxicity. The kidney is particularly susceptible to xenobiotics owing to its high blood supply and ability to concentrate toxins. Few studies have examined the impact of NPs in kidney, while both glomerular structures during plasma ultrafiltration and tubular epithelial cells may be exposed to NPs. Chen et al. [9] clearly observed damage to proximal tubular cells in mice exposed to copper NPs. Wang et al. [10] also observed signs of glomerulonephritis and pathological degeneration after oral titanium dioxide administration, within the renal proximal convoluted tubules. Additionally, recent bio-distribution studies confirmed NPs in kidneys and the influence of size or surface treatment on *in vivo *tissue distribution [11-13].

In the present studies, the effects of NP exposure on renal cells and their potential toxicity were investigated. The kidney is composed of different types of cells with varying sensitivities to toxic substances. Assays were conducted on two different cell lines (mesangial cell line, IP15 and proximal epithelial tubular cell line, LLC-PK_1_). These cell models were used by considering two important levels in nephrotoxicity. Mesangial cells are perivascular pericytes located within the central portion of the glomerular tuft between the capillary loops and are involved in the control of glomerular hemodynamics [14]. IP15 cells represent a human stable immortalized mesangial cell line and a suitable model to study *in vitro *cytotoxicity [15,16]. LLC-PK_1 _cells constitute an established cell line derived from normal pig kidney displaying several characteristics of the proximal tubule. This cell type is characterized by well-developed basal infolding and an apical brush-border, intense pinocytotic activity and variable transport or co-transport. They are also involved in intensive toxic accumulation [17].

Particle size, size distribution and dispersion in relevant biological media were first defined to assess toxicity accurately. NPs characterization in terms on the chemical properties was quantified in a Knudsen flow reactor using different probe gases that heterogeneously interact with the functional groups present on the NPs surface (Setyan, Sauvain and Rossi, personal publication submitted). Cellular morphology and mitochondrial function (WST-1 assay) for toxicity evaluations were assessed under controlled conditions. Cells were stained with phalloidin-FITC to detect of the cytoskeletal component, F-actin. To model the cellular uptake of particles, we used electron microscopy and modified fluorescent carboxylate and sulphate-modified polystyrene latex beads of various diameters. An additional study on oxidative stress was conducted using 2'–7'dichlorodihydrofluorescein diacetate (DCFH-DA) ROS assay. The mesangial and proximal tubular cell types were exposed to carbon black (CB) and to titanium dioxide (TiO_2_) particles with different average diameters. The data presented here constitute the first step towards the determination of a dose-effect correlation and a risk assessment of CB and TiO_2 _NPs on renal cells.

## Results

### Characterization of nanoparticles

Turbidity measurements performed to obtain the dispersion characterization of NPs (5 and 10 μg/cm^2 ^corresponding to 19.6 and 38.2 μg/ml) are presented in Table 1. Low values were obtained with CB NPs prepared either in RPMI 1640-serum free medium or in deionized water. No such NTU turbidity differences in RPMI were observed with TiO_2_. The difference in turbidity signal levels between CB and TiO_2 _must be imputed for carbon to the black colored suspension that it is necessary to take into account [18]. Complementary light microscopic analysis showed that NPs in RPMI 1640 were less but still aggregated. Therefore, electron microscopy observations were performed only with NPs prepared in the same culture medium as used for cell experiments.

**Table 1:** Physical characteristics of the nanoparticles

Nanoparticles powder	Diameter (nm)*	Source	BET surface (m^2 ^g^-1^)*	Crystal phase*	Turbidimetry (NTU) in H_2_0 [19.6 and 38.2 μg/ml]	Turbidimetry (NTU) in RPMI [19.6 and 38.2 μg/ml]	Average diameter (MET) nanoparticles [nm ± sd]	Aggregates diameter (MET) [Min – Max nm]
FW2	13	Degussa	350	Amorphous carbon	34 ± 1 61 ± 3	28 ± 3 51 ± 5	22,61 ± 5,93	[31–734]
P60	21	Degussa	115	Amorphous carbon	225 ± 10 432 ± 17	60 ± 2 107 ± 3	35,29 ± 10,06	[64–1891]
LB101	95	Degussa	20	Amorphous carbon	172 ± 7 286 ± 17	66 ± 3 116 ± 9	165,15 ± 62,02	[123–2804]
TiO_2_-15	15	Sigma Aldrich	200 – 220	99,7% metal basis 98% anatase/rutile**	158 ± 16 327 ± 32	173 ± 14 337 ± 28	11,69 ± 2,28	[67–1348]
TiO_2_-50	25 – 75	Sigma Aldrich	20 – 25	99,9% metal basis 65% anatase/rutile**	201 ± 4 397 ± 7	254 ± 6 500 ± 13	47,83 ± 32,97	[84–1364]

* data provided by the manufacturer

** data provided by Setyan et al.

Turbidimetry measurements were performed in RPMI 1640-serum free medium or in ultrapure deionized water to characterize particle dispersion rates at 19.6 and 38.2 μg/ml (corresponding to concentrations of 5 and 10 μg/cm^2^). Average diameter of nanoparticles, expressed as mean size ± SD nm, and average diameter of aggregates were determined using a transmission electron microscope fitted with a camera and SIS software. (*data provided by the manufacturer, **personal data, Setyan, Sauvain and Rossi submitted in Physical Chemistry Chemical Physics).

**Figure 1:**
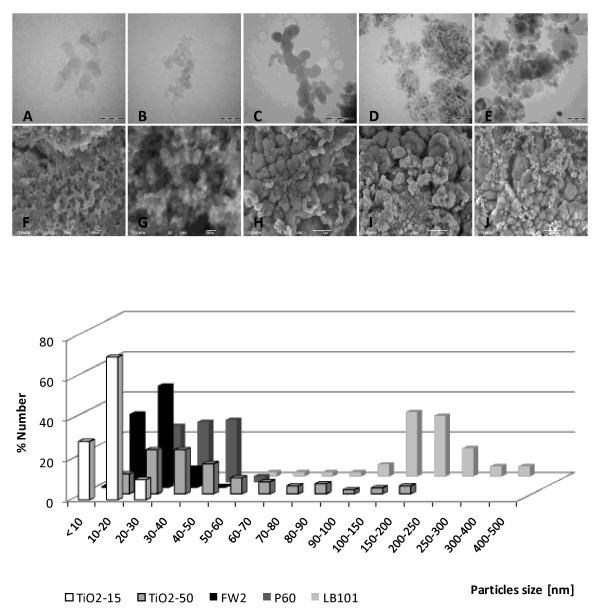
**Microscopy characterizations of NPs**. Transmission electron microscope (JEOL 2000 FX) and scanning electron microscope (JEOL 6700F High resolution) images of FW2 (A, F), P60 (B, G), LB101 (C, H), TiO_2_-15 (D, I) and TiO_2_-50 (E, J). NPs stock solutions (2 mg/ml) in RPMI 1640-serum free medium were prepared as described in Methods section (MET scale bars A: 50 nm, B D and E: 100 nm, C: 500 nm – MEB scale bars: F and G: 100 nm, H, I, and J: 1 μm). Distribution size histograms expressed as percentage were calculated using SIS software.

Transmission (Figures 1A–1E) and scanning electronic (Figures 1F–1J) microscopes were used to obtain photographs, diameter, size distribution and morphology information about NPs and aggregates. Experiments were performed using a 2 mg/ml stock suspension and a dilution of 19.6 μg/ml selected to coincide specifically with the 5 μg/cm^2 ^regularly tested. The mean particle size of CB was greater than that reported by the manufacturer, while mean TiO_2 _sizes were in agreement with the manufacturer-specified sizes (Figure 1). NPs in suspension not only led to distribution at the individual particle size with considerable polydispersity in LB101 and TiO_2_-50 solutions (Table 1). Most of the NPs exhibited a high level of agglomeration (mean ± se) reaching 188 ± 13 nm, 458 ± 64 nm and 1083 ± 100 nm for FW2, P60 and LB101, respectively. With TiO_2_, compact aggregates were observed (481 ± 48 nm, 1005 ± 270 nm for TiO_2_-15 and TiO_2_-50, respectively). These levels of agglomeration raise concerns when considering size dependent toxicity and dose dependent toxicity for *in vitro *experiments.

### Cytotoxicity

**Figure 2:**
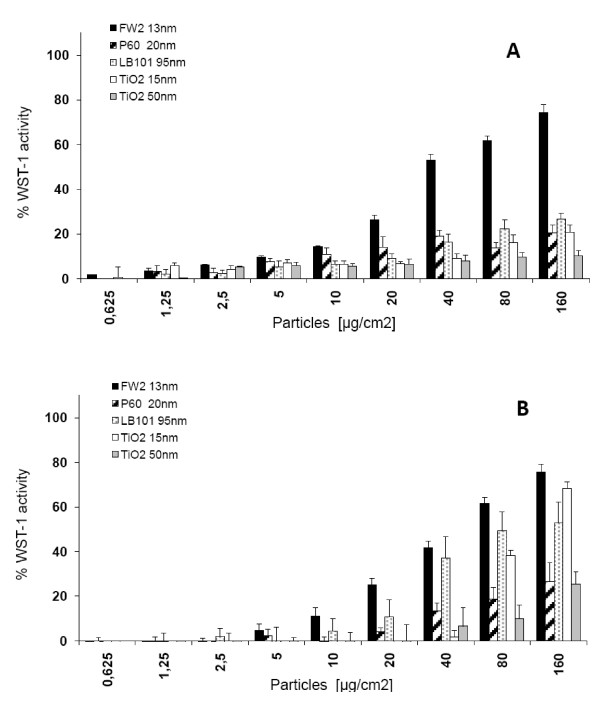
**Cytotoxicity of NPs**. Cytotoxicity effects of CB (FW2, P60 and LB101) and TiO_2 _(15 and 50 nm) NPs after 24 h exposure on (A) IP15 cells and (B) LLC-PK_1 _cells as assessed by WST-1 assay. Data were expressed as percentage of cell death related to untreated controls performed in the same experiment.

IP15 mesangial and LLC-PK_1 _tubular cells were exposed for 24 h to various CB and TiO_2 _NPs ranging from 0.625 to 160 μg/cm^2 ^concentrations in RPMI 1640-serum free medium. During the initial experiments, we observed apparent artifacts in the cytotoxicity concentrations measured (data not shown) using neutral red assay and we hypothesized that surface adsorption on CB particles was interfering with photometric readings of neutral red uptake. Thus, cytotoxicity experiments were conducted with the WST-1 assay. As shown in Figure 2A, the results showed that FW2 exhibited more toxicity on IP15 cells than other NPs. IC_50 _was calculated to be 30 μg/cm^2^. In contrast, P60 and LB101 had only a slight effect on mitochondrial function with 19.1 ± 2.4% and 16.3 ± 3.5% at 40 μg/cm^2 ^and 20.5 ± 3.5% and 26.7 ± 2.54% at 160 μg/cm^2^, respectively. Slight or no significant differences (21.0 ± 3.1% and 10.3 ± 2.3%) were also observed up to a concentration of 160 μg/cm^2 ^when using TiO_2_-15 and TiO_2_-50 NPs. The cytotoxicity index of the known toxic CdO was evaluated on IP15 cells to be 0.06 μg/cm^2^.

As shown in Figure 2B, cell sensitivity to NPs was different on LLC-PK_1 _cells. FW2-induced cell mortality appeared to be significantly increased above 10 μg/cm^2 ^with IC_50 _ranging from 40 to 60 μg/cm^2^. P60 did not induce any large toxic effects at concentrations up to 160 μg/cm^2 ^(26.6 ± 8.5%). On the other hand, LB101 induced a significant increase in cell mortality above 40 μg/cm^2^. Moreover, different sizes of nano-TiO_2 _exhibited different levels of toxicity on LLC-PK_1 _cells. The TiO_2_-15 particle was the most cytotoxic with an IC_50 _evaluated between 100–160 μg/cm^2^, while TiO_2_-50 showed only 25.3 ± 5.7% mortality at 160 μg/cm^2^. CdO used as positive control also produced toxic effects in LLC-PK_1 _with an IC_50 _evaluated around 0.5 μg/cm^2^.

### F-actin phalloidin

**Figure 3:**
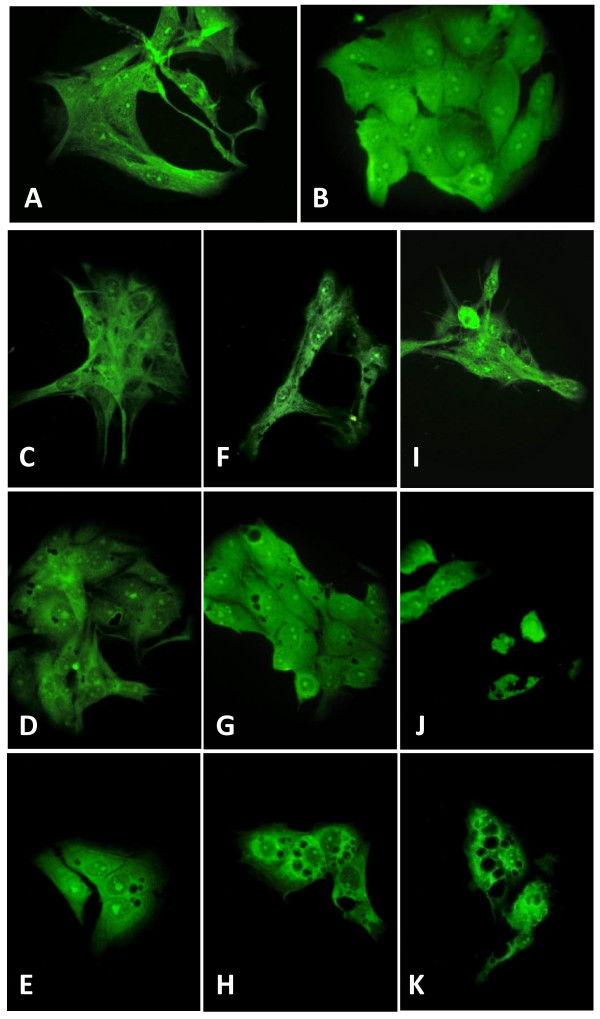
**Immunofluorescence labeling of F-actin after NPs exposure**. Representative micrographs using F-actin phalloidin FITC antibodies under epifluorescence microscope (Olympus BH2, magnification ×400) after 24 h exposure of NPs: control (A: IP15 cells, B: LLC-PK_1 _cells) – FW2 (C and D:1 μg/cm^2^, F and G:10 μg/cm^2 ^and I and J: 40 μg/cm^2 ^on IP15 and LLC-PK_1 _cells, respectively) – TiO_2_-15 (E: 1 μg/cm^2^, H: 10 μg/cm^2 ^and K: 40 μg/cm^2 ^on LLC-PK_1 _cells).

Because cytoskeleton microfilaments are involved in cell attachment and shape, the appearance of F-actin was observed after 24 h exposure to FW2 and TiO_2_-15 NPs, which are considered as highly cytotoxic. The fluorescence from control IP15 (Figure 3A) and LLC-PK_1 _(Figure 3B) cells was mainly distributed throughout the cells. At 1 μg/cm^2 ^of FW2 no distinct change in the morphology of IP15 (Figure 3C) and LLC-PK_1 _(Figure 3D) cells was observed. Similar micrograph was obtained with 1 μg/cm^2 ^of TiO_2_-15, as example on LLC-PK_1 _(Figure 3E). Figure 3F in IP15 cells and particularly Figures 3G and 3H in LLC-PK_1_, show vesicles inside the cell after 10 μg/cm^2 ^FW2 and TiO_2_-15 exposure. However, at 40 μg/cm^2 ^cells became abnormal in size, displaying cellular shrinkage and detachment from the surface of flasks in IP15 (Figure 3I) and in LLC-PK_1 _(Figures 3J, K). The cells appeared to have fewer cytoplasmic extensions.

### Latex beads

**Figure 4:**
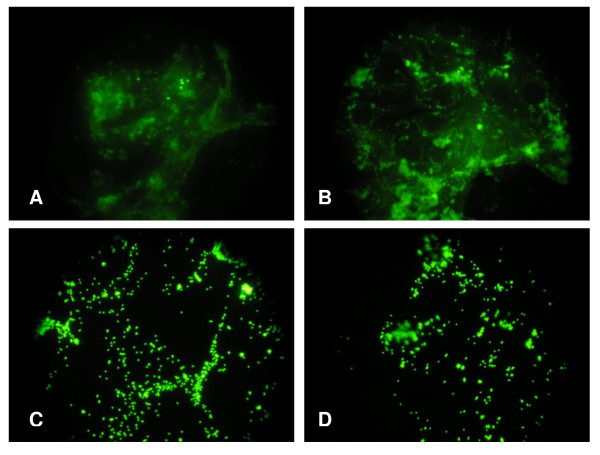
**Latex beads assay**. Micrographs of size-dependent internalization of fluorescently labeled polystyrene beads: 30 nm carboxylated-modified latex beads on IP15 cells (A) and LLC-PK_1 _cells (B), 500 nm sulphate-modified latex bead on IP15 cells (C) and LLC-PK_1 _cells (D) under epifluorescence microscope (Olympus BH2, magnification ×400).

Because cellular uptake is a necessary prerequisite, fluorescence microscopy was used qualitatively to determine the binding and uptake of latex beads (30 to 1000 nm in diameter) by the cells. Fluorescence-labeled latex beads were used directly and observed under microscopy from the cell layers. In Figure 4 showing IP15 and LLC-PK_1 _cells stained by 30 nm fluorescent latex beads (Figures 4A, B) most of the fluorescent particles were internalized by the cells. No internalization of the 500 nm (Figures 4C, D) and 1000 nm (micrographs not shown) latex beads was observed.

### NPs uptake by TEM

**Figure 5:**
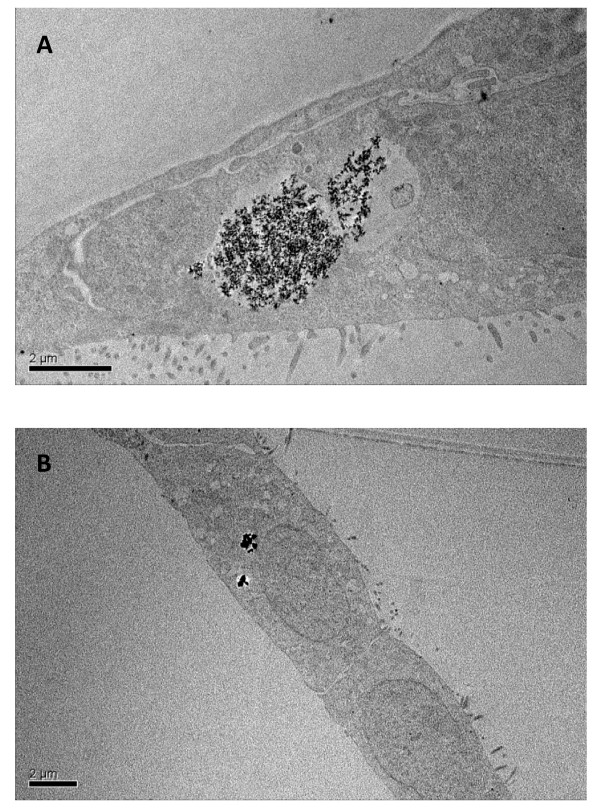
**Transmission electronic microscopy observations of NPs uptake**. Transmission electronic microscope micrographs of LLC-PK_1 _cells which internalized FW2 (A) and TiO_2_-15 (B) NPs (MET scale bars A and B: 2 μm, images were taken at ×14 000 and 10 000 magnification).

Electron micrographs show the well differentiated morphology of the LLC-PK_1 _cell line particularly the well developed brush border (Figure 5). NPs FW2 (Figure 5A) and TiO_2_-15 (Figure 5B) were incorporated into the cells and often present within cytoplasmic vesicles.

### ROS DCFH-DA assay

**Figure 6:**
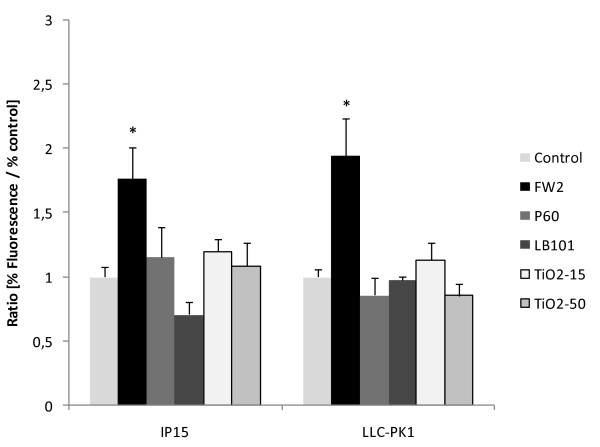
**ROS production after NPS exposure**. Effects of 5 μg/cm^2 ^of CB (FW2, P60 and LB101) and TiO_2 _(15 and 50 nm) NPs on the oxidation of DFCH-DA to DCF in IP15 cells and LLC-PK_1 _cells, after 6 h. Data represent the mean ± SE of fluorescence ratios (fluorescence of exposed cells/fluorescence of unexposed cells controls). *p < 0.01 indicates significant differences compared to control.

To investigate the potential role of oxidative stress as a mechanism of NP toxicity, a DCFH-DA assay was performed after 4, 6 and 24 h of NPs exposure. ROS levels peaked at 6 h and then diminished over a period of 24 h with FW2 (data not shown). Therefore, ROS generation was expressed only at 6 h exposure to different CB and TiO_2 _at a concentration of 5 μg/cm^2 ^(Figure 6). FW2 induced a significant increase in DCF fluorescence as expressed as by fluorescence ratio on both cell lines. Neither P60 and LB101 nor TiO_2 _induced significant changes.

## Discussion

NPs have been shown to reach the systemic circulation after inhalation, ingestion or intravenous injection, with further distribution to several organs such as liver, spleen, kidneys, brain or heart [4,19-21]. However, particle size clearly affects their distribution, as suggested by De Jong et al. [22]. Indeed, after intravenous injection in the rat tail vein, systemic distribution was evidenced for gold NPs (10 nm diameter), with further detection in various organs including liver, spleen, kidney, testis, thymus, heart, lung and brain. Larger particles were also systemically distributed, but detected in the liver and spleen only. This is in accordance with recent bio-distribution experiments showing NPs in the kidneys [10,11,23]. Wang et al., observed damage of kidneys after TiO_2 _single oral gavage due to the small size and particle clearance [10]. Moreover, exposition to copper NPs clearly led to renal proximal tubular cell damage in mice, with swollen glomeruli reflecting glomerulonephritis, altered blood biochemical indexes, in particular noticeable changes in urea nitrogen, creatinine and alkaline phosphatase levels, all suggesting altered renal functions [9]. Others studies suggested that nano-drugs could be targeted and incorporated by renal glomerular mesangial cells [24,25]. Meanwhile, short- or long-term impacts on renal functions after NPs accumulation in the kidneys have not been assessed yet, and, to our knowledge, few *in vitro *data are currently available on NPs toxicity in renal cells.

In this study, we evaluated the potential *in vitro *effects of different commercially available NPs (carbon black -FW2, P60 and LB101- or TiO_2 _nanoparticles) on glomerular mesangial cells (IP15) or epithelial proximal tubular cells (LLC-PK_1_). We have previously evaluated and validated the use of such cell lines to assess renal toxicity [16,26,27]. Therefore, developing such *in vitro *models to assess NPs renal toxicity would be of particular interest regarding development of routine screening tests and investigation of NPs precise mechanisms of action.

First, we analyzed NPs by electronic microscopy and turbidimetry under conditions close to toxicity experiments in the RPMI 1640- free serum medium. We showed that these particles do not necessarily retain their nano-size in solution. Indeed, carbon black as well as TiO_2 _particles tended to aggregate into larger complexes, with for example an 8-, 13- or 6 fold-increased size for FW2, P60 or LB101 aggregates respectively, compared to the initial size of isolated particles.

Measuring the viability of cells, by neutral red or LDH assay after chemical exposition is a routinely made method to assess toxicity information. Another standard assay used for analysis is the measurement of the activity of the mitochondrial dehydrogenases by formation of a formazan product which is water soluble in the WST-1 assay in contrast to the formazan product generated in the MTT assay. Carbon black particles can adsorbed substances such as neutral red or MTT [28-30]. We confirmed this result in some of our experiments, showing interferences caused by these particles in the neutral red cytotoxicity assay. Keeping in mind these interactions, all of our microscopy characterization and cytotoxicity assays were therefore conducted in a red-phenol free medium without FBS supplementation. We assessed NPs potential cytotoxicity by use of the WST-1 assay; the WST-1 assay has been validated in various *in vitro *toxicity assays using such particles [31,32].

*In vitro *cytotoxicity assays conducted in this study highlighted different responses in function of the nanoparticle tested and/or the cell type considered. On both IP15 and LLC-PK_1 _cells, the smallest FW2 NP was found to be the most cytotoxic with classic dose-behavior. For the other NPs tested (LB101 and TiO_2_), different cytotoxic profiles were found, with LLC-PK_1 _cells being more sensitive than IP15 cells. On IP15 cells, CB LB101, P60 and TiO_2 _displayed less or no toxicity at the doses tested. On LLC-PK_1 _cells, the cytotoxic response of LB101 was different and size-dependent for TiO_2_. Previous results showed the ability of NPs to induce various effects in function of the cell type considered. For example, Patra et al. reported a gold nanoparticle-induced death response in a human carcinoma lung cell line (A549 cells) whereas no effect was observed in BHK21 (hamster kidney) or HepG2 (human hepatocellular liver carcinoma) cells [33]. Accordingly, such differences were observed in our experiments for example with TiO_2 _particles, inducing cell death in LLC-PK_1 _but not in IP15 cells. The degree of cytotoxicity was correlated to the particle size, in accordance with numerous studies showing that smaller TiO_2 _and CB particles are more toxic than larger ones [1,34-36]. However, apart from size, other structural characteristics may also participate in the various cytotoxic effects observed when studying different cell types. For example, Warheit et al. demonstrated that crystal structure and surface chemical reactivity may also be involved [13]. In particular, chemical reactive groups present at the surface of the particles may differ from one particle type to another as recently suggested by our group (Setyan et al., personal publication, submitted), therefore leading to various cytotoxic profiles when interacting with different cell types,

An unsolved issue is whether nanoparticles are internalized into cells, and if so, which internalization mechanisms are involved. To assess this question, we first conducted experiments to evaluate whether fluorescent latex beads may be internalized in both cell types. We therefore showed that small particles (30 nm) were internalized into the cell cytoplasm, whereas larger particles (500 nm) remained outside the cells. This result therefore demonstrates that particle internalization is possible in these cells, but is size-dependent. However, the size of the latex beads internalized is consistent with the size of our NPs of interest, suggesting a possible internalization mechanism for carbon black -FW2, P60 and LB101- or TiO_2 _NPs. Apart from particle size, other features such as anionic or cationic groups on surface, in particular by use of carboxylate or sulphate polystyrene latex beads, may also affect particles internalization. This will be investigated in the future. F-actin fluorescent microscopy images revealed an increase of cell vacuoles and electronic microscopy images show NPs trapped in these vacuoles. Single particles were no or rarely observed inside the cytoplasmic cell, perhaps due to their small size. Internalization seemed to be more important in LLC-PK_1 _cells. These epithelial cells are polarized with a typical apical brush border membrane and also display an intense endocytic activity; these characteristics may therefore participate in enhanced NPs internalization in these cells compared to IP15 cells, and may therefore take part in the enhanced cytotoxicity observed in LLC-PK_1 _cells.

ROS generation was investigated thereafter by use of the DCFH-DA assay. For this study, the oxidation of DCFH was chosen on the basis of the reactivity of this molecule with different ROS including H_2_O_2 _and superoxide. In the literature, various NPs have been demonstrated to generate ROS and to a greater extent more than larger particles, and this mechanism is thought to play a role in their cytotoxic effects [37,38]. In our experiments, no generation of ROS was evidenced in any cell type after treatment with P60, LB101 or TiO_2 _particles. By contrast, exposure to FW2 NP, evidenced in our previous experiments as the most cytotoxic particle type, significantly enhanced production of ROS in both IP15 and LLC-PK_1 _cells. The precise mechanisms activated by NPs to induce ROS generation and induce cytotoxicity are not known yet. NPs may activate intracellular pathways involving in particular increase in calcium intracellular concentrations [39,40], involvement of MAPK [41,42], and activation of transcription factors leading in particular to synthesis of pro-inflammatory cytokines and chemokines [1,36]. In addition, when particles enter the cell, they could induce oxidative stress by disturbing the balance between oxidant and anti-oxidant processes, as for example the glutathione system. In epithelial cell line, Stone et al. [43], demonstrated that ultrafine carbon can induce glutathione depletion. The signaling mechanisms activated by such nanoparticles to induce cytotoxicity, in particular through generation of reactive oxygen species, therefore merit further attention.

## Conclusion

In this work, we described for the first time the effects of CB and TiO_2_nanoparticles in renal cells *in vitro*. These effects may have relevance *in vivo *considering the ability of nanoparticles to reach the systemic circulation and to be further distributed to numerous organs, in particular to the kidneys. We described the ability of these nanoparticles to exert a cytotoxic effect on renal cells, and suggested involvement of particle internalization as well as activation of intracellular mechanisms that might include generation of reactive oxygen species. However, we showed that cytotoxicity was particle type-, particle size- and cell type-dependent. Moreover, the doses of nanoparticles that were cytotoxic *in vitro *correspond to a concentration of 20 μg/cm^2^, which may not be physiologically relevant. However, these results raised numerous questions regarding NPs long term secondary effects, bioavailability and precise toxicological effects, in particular in renal cells. The further development of *in vitro *models, in particular cell culture models, their validation regarding translation of the results obtained *in vitro *to *in vivo *models and their standardization will be of great help to assess these questions and investigate the nanoparticle precise mechanisms of action to induce cell toxicity.

## Methods

### Chemicals

The particles used in these experiments were CB NPs (FW2 color black, P60 pigment black Printex^®^60, LB101 Lamp black) purchased from Degussa (Dusseldorf, Germany) and TiO_2 _obtained from Sigma Aldrich (St-Quentin-Fallavier, France). The particle composition as amorphous carbon was identical for all types of CB. However, diameters and surface areas varied. Manufacturer-specified information is summarized in Table 1. All chemicals were used "as received" without further purification. As a positive control for toxicity, we used cadmium oxide (CdO), a large-sized material (1 μm) known for its cytotoxic properties (Fluka Chemicals, St-Quentin-Fallavier, France).

All products used for cell culture were purchased from Lonza (Verviers, Belgium) (exceptions mentioned). RPMI 1640 without neutral red, HBSS (Hank's Balanced Salt Solution), PBS (Phosphate Buffer Saline) used for ROS and cytotoxicity assay were also purchased from Lonza (Verviers, Belgium). WST-1 was from Roche Diagnostics (Meylan, France). Dichlorodihydrofluorescein diacetate (DCFH-DA) for ROS and carboxylate-modified (30 nm, and 1000 nm) and sulphate-modified (500 nm) polystyrene latex beads for fluorescence microscopy were from Sigma Aldrich (St-Quentin-Fallavier, France).

### Preparation and characterization of particles

Stock suspensions (2 mg/ml) of each nanoparticle and CdO were prepared in RPMI 1640-serum free medium without neutral red or in ultrapure deionized water by brief sonication (20 s, 9 times) (Vibracell 75186, 130 W, 56–60 Hrz) and frozen immediately. Prior to each cell culture experiment, stock solutions were again suspended by sonication (20 s, 3 times). Sonication was utilized to facilitate particle dispersion and solution mixture.

NPs characterization was performed using a transmission electron microscope TEM (JEOL 2000FX). NPs were examined after suspension in biological media and subsequent deposition onto collodion-coated carbon grids. SIS software for the TEM camera was calibrated to measure the size of NPs. Particle morphology was also observed using a high resolution scanning electron microscope SEM (JEOL 6700F) operated at 5 Kv.

Turbidimetry measurements of NPs prepared in RPMI 1640-serum free medium or in ultrapure deionized water were used to characterize particle dispersion rates. Turbidity measurements were carried out using a HACH 2100AN turbidimeter which includes a tungsten-filament lamp and a 90° and 180° light detectors to monitor scattered and transmitted light. Turbidity, expressed in Nephelometric Turbidity Units (NTU), quantifies the degree to which light travelling through a sample is scattered by the suspended particles.

### Cell cultures

Human IP15 mesangial cells, a gift from Dr. I. Dubus (Department of Biochemistry, University Bordeaux 2, France), were cultured in RPMI 1640 medium containing penicillin (100 U/ml), streptomycin (100 μg/ml) and amphotericin B (0.25 μg/ml), 2 mM L-glutamine, sodium pyruvate, non-essential aminoacids and 10 mM Hepes supplemented with 10% inactivated fetal bovine serum (FBS) (Eurobio, Les Ullis, France). LLC-PK_1 _cells derived from the Hampshire pig were purchased from the European Collection of Cell Cultures (ECACC) and used between the 210^th ^and 245^th ^passages. Cells were grown in EMEM (Eagles Minimum Essential Medium) supplemented with 10 mM Hepes, 2 mM L-glutamine, streptomycin (100 U/ml) and penicillin (100 μg/ml) supplemented with 5% FBS. Both cell cultures grew in 75-cm^2 ^plastic culture flasks (Greiner BioOne, Courtaboeuf, France) and were maintained in 5% CO_2 _– 95% air atmosphere. The media were changed every 2 days and cells were trypsinized when necessary (0.05% trypsin – 0.53 mM EDTA).

### Cytotoxicity assay

Mitochondrial activity was assessed with the WST-1 assay based on cleavage of the water soluble tetrazolium salt to a formazan dye by succinate-tetrazolium reductase, which exists in the mitochondrial respiratory chain and is active only in viable cells.

Cells grown in 96-well plates, seeded at 50 000 cells/cm^2 ^for LLC-PK_1 _and 65 000 cells/cm^2 ^for IP15 cells, were used at subconfluence (24 h) and exposed (100 μl) to varying concentrations of CB, TiO_2 _particles and CdO. In relation to the cell surface dishes used (0.32 cm^2^/well), different concentrations ranging from 2 to 512 μg/ml were prepared and corresponded from 0.625 to 160 μg/cm^2^.

Cells were first washed with RPMI 1640-serum free medium and NPs were added for 24 h. After exposure, cells were washed and incubated at 37°C for an additional 30 min (for IP15 cells) or 1 h (for LLC-PK_1_) in culture medium containing the WST-1 solution (10 μl/well). The quantity of formazan dye was determined with a photometer at 450 nm compared to a 630 nm reference. The data from at least 3 independent triplicates were expressed as percentage of dead cells compared to a control from the same experiment.

### Immunofluorescence Phalloidin-FITC assay

This study was designed to determine whether NPs induce morphological changes of the actin cytoskeleton. The intracellular organization was visualized by staining FITC-conjugated phalloidin. Cells were grown on glass slides to subconfluence and treated at appropriate concentrations of NPs (1, 10, and 40 μg/cm^2^). After exposure, cells were fixed, permeabilized with ethanol and then incubated for 1 h with phalloidin-FITC (10 μM). Cytoskeleton elements were visualized using the epifluorescence microscope (Olympus BH2, Rungis, France).

### Fluorescence latex beads

Cells were seeded on glass slides (Lab-Teck Falcon, Becton Dickinson, Meylan, France) and used at subconfluence. The media was replaced with a 2.5% latex bead suspension at a final dilution of 0.0085% (equivalent to 50 μg/cm^2^) in RPMI 1640-serum free medium for 6 h. After additional washing, cells were fixed with ethanol and the green fluorescence of carboxylate-modified (30 nm and 1000 nm) and sulphate-modified (500 nm) polystyrene latex beads was visualized under an epifluorescence microscope (Olympus BH2, Rungis, France).

### NPs uptake by TEM

LLC-PK1 cells in 75-cm2 plastic culture flasks were used at subconfluence. Cells exposed to 5 μg/cm^2 ^of FW2 and TiO2–15 for 24 h were washed with PBS, fixed by 2.5% glutaraldehyde in 0.045 M sodium cacodylate buffer at 4°C for 2 hours and postfixed in 1% osmium tetroxide (pH 7.4). The cells were scraped off and washed with sodium cacodylate buffer. After dehydration in ascending grades of ethanol, cells were subsequently embedded in epoxy resin. Ultrathin sections (60 nm) were performed using ultramicrotome before observation with a JEOL1200 EXII electron microscope.

### Reactive Oxygen Species (ROS) assay

The generation of ROS in serum-free media was determined with the 2'–7' dichlorodihydrofluorescein diacetate (DCFH-DA) reagent as described by Canal-Raffin [44]. Briefly, after cells became subconfluent in 60-mm Petri dishes (19.6 cm^2^), cells were incubated 15 min with 10 μM or 50 μM of DCFH-DA for IP15 and LLC-PK_1_, respectively. DCFH-DA is a stable, non-fluorescent molecule that is hydrolyzed by intracellular esterases to non-fluorescent 2', 7' -dichlorofluorescein (DCFH), which is rapidly oxidized in the presence of peroxides to a highly fluorescent adduct [45]. Cells were washed with PBS and treated with different concentrations of NPs (5 μg/cm^2^) for 6 h. After exposure, the cells were scraped off, lysed by sonication and centrifuged. Supernatants were collected and ROS levels were determined at excitation wavelength 488 nm and emission wavelength 520 nm using a fluorimeter (Kontrol Instrument, SFM 25, Eching, Germany). Data from at least 3 independent triplicates are reported as fluorescence intensity percentage and expressed as mean fluorescence ratio (fluorescence of exposed cells/fluorescence of unexposed control from the same experiment).

### Statistical Analysis

TEM information on mean size ± SD, using SIS software was calculated by measuring over 50 NPs in random fields of view in addition to images showing the morphology of the NPs. For cytotoxicity experiments, results were calculated using the formula (100 – (Absorbance treated sample × 100/Absorbance control sample)) and expressed as mean ± SE. Non-linear Boltzman regression analysis was performed using the Origin^® ^software (Origin Lab. Corp, Northampton, USA) and the IC_50 _(defined as concentration which induces 50% cell viability decrease) were calculated. For the DCFH-DA assay, data were expressed as mean fluorescence ratio ± SE of at least three independent experiments. Statistical analysis was carried out by analysis of variance (ANOVA) and comparison of means was performed using Student's t-test. For all experiments, *p values < 0.01 was considered as significant.

## Competing interests

The authors declare that they have no competing interests.

## Authors' contributions

BL planned the study and contributed to all the sections; JJ carried out the cytotoxicity, ROS and immunofluorescent labeling studies. ES performed SEM and TEM nanoparticles analyses. DO and FM helped to prepare all cell specimens for electron microscopy. JF performed TEM observations. JC and PB provided advice and infrastructure. The study design and preparation of the manuscript were by COC.
